# The role of self-esteem in the development of psychiatric problems: a three-year prospective study in a clinical sample of adolescents

**DOI:** 10.1186/s13034-017-0207-y

**Published:** 2017-12-29

**Authors:** Ingvild Oxås Henriksen, Ingunn Ranøyen, Marit Sæbø Indredavik, Frode Stenseng

**Affiliations:** 1 Regional Centre for Child and Youth Mental Health and Child Welfare, Faculty of Medicine, NTNU, Trondheim, Norway; 2 Department of Child and Adolescent Psychiatry, St. Olavs Hospital, Trondheim University Hospital, Trondheim, Norway; 3 Queen Maud University College, Trondheim, Norway

**Keywords:** Mental health, Identity, Resilience, Internalizing and externalizing problems, Structural equation modeling

## Abstract

**Background:**

Self-esteem is fundamentally linked to mental health, but its’ role in trajectories of psychiatric problems is unclear. In particular, few studies have addressed the role of self-esteem in the development of attention problems. Hence, we examined the role of global self-esteem in the development of symptoms of anxiety/depression and attention problems, simultaneously, in a clinical sample of adolescents while accounting for gender, therapy, and medication.

**Methods:**

Longitudinal data were obtained from a sample of 201 adolescents—aged 13–18—referred to the Department of Child and Adolescent Psychiatry in Trondheim, Norway. In the baseline study, self-esteem, and symptoms of anxiety/depression and attention problems were measured by means of self-report. Participants were reassessed 3 years later, with a participation rate of 77% in the clinical sample.

**Results:**

Analyses showed that high self-esteem at baseline predicted fewer symptoms of both anxiety/depression *and* attention problems 3 years later after controlling for prior symptom levels, gender, therapy (or not), and medication.

**Conclusions:**

Results highlight the relevance of global self-esteem in the clinical practice, not only with regard to emotional problems, but also to attention problems. Implications for clinicians, parents, and others are discussed.

## Background

*Self*-*esteem*—in its broadest sense—is how much value a person place on his or herself [1]. Self-esteem is related to a person’s ability to hold a favorable attitude towards one self [2], and to retain such positive beliefs in situations that are challenging, especially situations that include being evaluated by others [3, 4]. Adults possessing high global self-esteem are more likely to have e.g. higher well-being, better social relations, and experience more job satisfaction than their counterparts [5]. Low self-esteem is related to e.g. emotional problems, substance abuse, and eating disorders [6]. Although self-esteem is regarded as a rather stable part of personality, it also fluctuates dependent on recent fails or accomplishments [7, 8], and sublevels of self-esteem also exists in relation to particular domains of one’s life, such as sports and spare time activities [9, 10].

Perhaps due to its idiosyncratic nature, the concept of self-esteem has been widely debated in the psychological literature [1, 11, 12]. Nevertheless, in spite of its unsettled definition, the concept of self-esteem has been extensively studied, and in particular in community samples. It has been widely studied in relation to subjective well-being and quality of life, and in domains such as schools, work, and sport activities [1, 13]. Meanwhile, few researchers have investigated the potential protective role of self-esteem in the development of psychiatric problems in adolescence. Hence, the role of self-esteem in the development of psychiatric conditions is largely unknown.

In the present study, then, based on 3-year longitudinal data on adolescents with psychiatric problems, we examined the potential protective role of self-esteem on later development of psychiatric problems. Before we turn to the empirical part of this report, we review studies relevant to this scope.

As mentioned above, several studies have explored the relationship between self-esteem and psychological outcomes in community samples. For example, Greenberg et al. [10] found that high self-esteem had an anxiety-buffering function among students in an experimental setting. Likewise, threats to self-esteem have been shown to induce anxiety [14, 15] and to activate strategies that defend or restore a person’s self-esteem [16]. In a longitudinal study, including nearly 3000 participants from two samples aged 15–21 years, Orth, Robins and Roberts [17] showed that low self-esteem more strongly predicted depression, than depression predicted low self-esteem. Moreover, a large meta-analysis by Sowislo and Orth [18], comprising a total of 85 longitudinal studies, concluded that the effect of low self-esteem on negative affectivity is solid and holds across different samples and design characteristics of studies, but notably, mostly limited to community samples. This corresponds with a review by Orth and Robins [19], concluding that there is massive empirical evidence in support of the vulnerability hypothesis of the *self*-*esteem and depression link*, which suggests that low self-esteem contributes to depression, and not vice versa. In other words, high self-esteem seems to play a protective role in the development of poor mental health, perhaps through higher levels of self-efficacy and better coping mechanisms [20, 21] but studies on clinical samples are lacking.

The majority of research on self-esteem and mental health has focused on internalizing problems, but it is also plausible to suggest that self-esteem may be related to externalizing problems, such as attention-deficit/hyperactivity disorder (ADHD). Impulsivity, inattention, and hyperactivity are core symptoms of ADHD, and the disorder is associated with impairments in social, emotional, academic, and behavioral domains [22]. Although there is some controversy linked to the onset of ADHD [23], symptoms often becomes evident in early childhood and persist throughout adulthood [24, 25]. It has been shown that self-esteem is lower among children with ADHD than children without the diagnosis [26, 27], and untreated ADHD is associated with low global self-esteem [28]. In a clinical study, Slomkowski, Klein, and Mannuzza [29] found that adolescents with ADHD who reported higher than average self-esteem reported fewer symptoms, indicating a protective role of self-esteem in the development of ADHD symptoms. Indeed, higher self-esteem and better social adjustment are considered important treatment targets for children with ADHD [28]. Nevertheless, the exact role of self-esteem in trajectories of longer-term attention problems is unclear.

In sum, self-esteem has been explored in a great number of studies conducted in community samples, and results indicate that low self-esteem may increase negative affectivity and anxiety. However, with regards to behavior problems, such as ADHD, results are inconclusive. To the best of our knowledge, virtually no studies have investigated the potential protective role of self-esteem on the development of attention problems and symptoms of anxiety/depression among adolescents in a clinical psychiatric setting. We approach this subject through a semi-reciprocal longitudinal model, with the aim of contributing to enhanced understanding of the relationship between self-esteem and mental health.

The following main hypotheses were stipulated in this study:

1. Self-esteem protects against the development of more anxiety/depression symptoms in a clinical psychiatric sample of adolescents.
2. Self-esteem protects against the development of more attention problems, but to a lesser extent than for internalizing problems (anxiety and depression symptoms).
3. Self-esteem is negatively correlated to both anxiety/depression symptoms and attention problems in a clinical psychiatric sample of adolescents.

## Methods

### Study design

The study is part of The Health Survey in the Department of Child and Adolescent Psychiatry (CAP), St. Olavs Hospital, Trondheim University Hospital, Norway. This clinic provides diagnostic assessment and treatment for all psychiatric conditions in referred children and adolescents, aged 0–18 years. This was a prospective study of a defined clinical population. Inclusion criteria in the baseline study were: referred adolescents, aged 13–18 years, who had at least one personal attendance at the clinic between February 2009 and February 2011. Exclusion criteria were: major difficulties in answering the questionnaire due to their psychiatric state, cognitive function, visual impairments, or lack of sufficient language skills. Emergency patients were invited to take part once they entered a stable phase. Follow-up of participants was conducted from 2012 to 2014, approximately 3 years after their first assessment, depending on the time for their first visit at the clinic Participation in the follow-up study did not require attendance at the CAP clinic.

### Study procedure

Newly referred patients as well as patients already enrolled at the CAP clinic received oral and written invitations at their first attendance after the project started. Written informed consent was obtained from adolescents and parents prior to inclusion, according to the CAP survey procedures. Relevant for this study: the participating adolescents responded to an electronic questionnaire about his or her mental and physical health in conjunction with an appointment at the clinic, without the presence of their parents. The questionnaire was accessed via a password-protected website. A project coordinator provided assistance if needed. Participants had a unique ID-code linked to their questionnaire. Once the questionnaire was submitted, it was not possible to resubmit a new questionnaire using the same code. In addition, data were collected from clinical charts. At follow-up, adolescents from baseline were invited to respond to an electronic questionnaire measuring physical and mental health status, using the same ID-code.

### Study population

**Fig. 1:**
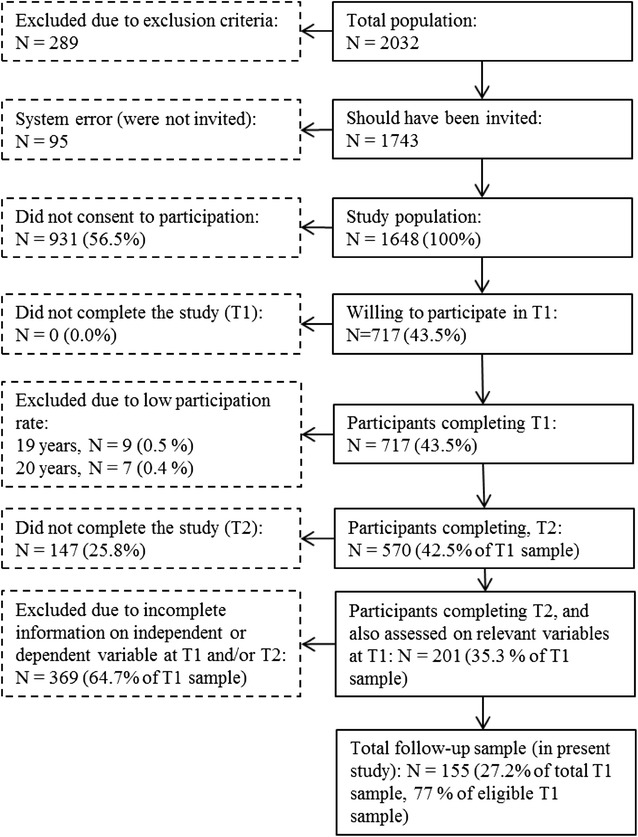
Flow chart of the recruitment and attrition in the present study

In the first study period, 2032 adolescent patients had at least one attendance at the CAP clinic. Of these, 289 were excluded on the basis of the exclusion criteria. Also, 95 were lost to registration (missing). Inclusion criteria were: adolescents aged 13–18 years, who had at least one personal attendance at the clinic over a 2-year period (February 15, 2009 to February 15, 2011). Exclusion criteria were: major difficulties in answering the questionnaire due to their psychiatric state, cognitive function, visual impairments or lack of sufficient language skills. Emergency patients were invited to take part once they entered a stable phase. Hence, 1648 patients (81.1%) were invited to participate. Of these, a total of 717 adolescents (43.5%), aged 13–18 years, participated in the baseline CAP survey; 393 girls (54.8%) and 324 boys (45.2%). All baseline participants, who had consented to being contacted for follow-up (*n* = 685), by then aged 16–21 years, were invited. Among the invited 570 participated (83%) at follow-up: 324 girls (57%) and 246 boys (43%). Mean birth year of participants was 1994. Mean age was 15.66 years (*SD* = 1.65). To explore the representativeness of the baseline study population, anonymous information about the reference population was collected from annual reports from St. Olav’s University Hospital, 2009–2011. All adolescents in the study period (*N* = 2032) minus those excluded (*n* = 289) were defined as reference population (*n* = 1743). In accordance with the permission given by the Norwegian Social Science Data Services, Data Protection Official for Research, we compared age, sex, and main reason for referral between participants (*n* = 717) and non-participants (*n* = 1026) of the reference population. Participants were 0.27 years older, 95% CI (.10, .45), than non-participants, *M* = 15.66, *SD* = 1.65 versus *M* = 15.39, *SD* = 1.95, *p* = .002. There were more girls in the study group than in the non-participating group, 393 girls (54.8%) versus 509 girls (49.6%), *p* = .032. Main reason for referral did not differ between participants and non-participants (Pearson exact Chi square test; *p* = .11). Five hundred and ninety-four of these participants (86.5%) received therapy at T1, and 278 participants (40.5%) received medication. Of the 570 participating at follow-up, 201 subjects (122 girls, 61%, and 79 boys, 39%), had been assessed for attention problems and/or emotional problems at baseline, and thus constitute the sample of the present study. Of these 201 eligible participants from T1, a total of 155 participants responded to all study variables in T2, 96 girls (62%) and 59 boys (38%), which corresponds to a participation rate of 77% (see Fig. 1) in the clinical sample.

### Ethics

At both baseline and follow-up, written informed consent was obtained from the adolescents and parents prior to inclusion and from the parents of participants younger than 16 years of age, according to the study procedures in the CAP survey. Study approval was given by the Regional Committee for Medical and Health Research Ethics (reference numbers CAP survey T1: 4.2008.1393, T2: 2011/1435/REK Midt; present study: 2015/845/REK Midt), and by the Norwegian Social Science Data Services (reference number CAP survey: 19976).

### Measures

#### Self-esteem

The Rosenberg Self-Esteem Scale [2] (RSES) is a Likert-type scale with items answered by self-report on a 4-point scale (1 = *strongly agree*, 4 = *strongly agree*). In the present study, self-esteem was scored on a scale ranging from 4 to 16 using a short version of the RSES, consisting of four statements: “I take a positive attitude towards myself”; “I feel I am a valuable person, at least on par with others”; “I really feel useless at times”; and “I feel I do not have much to be proud of”. Scores on negative phrases were inverted. The RSES has exhibited high validity in several studies [30–32] and is widely used across nations in exploring self-esteem [33]. Cronbach’s alpha was .85.

#### Anxiety/depression and attention problems

The Youth Self-Report [34] (YSR) is a part of the Achenbach System of Empirically Based Assessment. It provides self-rating on 112 problem items. Each item is rated on a scale of 0–2 (0 = *not true*, 1 = *somewhat or sometimes true*, 2 = *very true or often true*). The problem checklist contains eight core syndrome scales [34]. In this study, the syndrome scales anxious/depressed and attention problems were used. Baseline YSR was collected from clinical charts of those participants who had responded to YSR as part of the clinicians’ diagnostic evaluation. At follow-up the YSR was obtained directly by the Hel-BUP project as the YSR was incorporated in the questionnaire answered by all participants. The study population for this particular study consists of participants who answered YSR both at baseline and follow-up.

## Results

### Descriptive analyses

Descriptive analyses were performed in SPSS Version 21. Mean values and standard deviations for study variables are presented in Table 1. Mean level of self-esteem in the total sample was 9.41 (*SD* = 3.08) at baseline. Symptoms of anxiety/depression significantly decreased from 8.92 (*SD* = 6.39) at baseline to 7.44 (*SD* = 5.95) at follow-up. Additionally, mean levels of attention problems decreased from 7.83 (*SD* = 3.87) at baseline to 6.80 (*SD* = 3.70) at follow-up.

**Table 1:** Correlations, mean values, and standard deviations among study variables at baseline (T1) and follow-up (T2)

	1	2	3	4	5	6	7	8	Mean	SD
1. Birth year	1								1994.2	1.572
2. Gender^a^	.188*	1							.38	0.487
3. Therapy^b^	− .275**	− .212**	1						.88	.32
4. Medication^c^	− .198*	.170*	.299**	1					.40	.49
5. Self-esteem T1	.106	.477**	− .116	.126	1				9.413	3.079
6. Anxious/depressed T1	− .151	− .451**	.201*	− .072	− .583**	1			8.916	6.393
7. Attention problems T1	− .136	− .184*	.169*	.165*	− .331**	.410**	1		7.832	3.871
8. Anxious/depressed T2	− .146	− .220**	− .334**	.298**	− .566**	.608**	.328**	1	7.439	5.954
9. Attention problems T2	− .148	− .229**	.143	.239**	− .332**	.300**	.564**	.540**	6.800	3.702

*p < .05, **p < .01

^a^Boy = 1, Girl = 2; ^b^ 1 = No, 2 = Yes; ^c^ 1 = No, 2 = Yes

### Correlation analysis

There were significant negative correlations between self-esteem and symptoms of anxiety/depression and attention problems (see Table 1) at baseline. There was a strong positive correlation between symptoms of anxiety/depression at baseline and at follow-up. Similarly, the correlation between attention problems at baseline and follow-up was moderately significant. Anxiety/depression at baseline was positively correlated with attention problems, both at baseline and at follow-up. The cross-time correlation between psychiatric problems at baseline and follow-up was significant for both categories of problems. There was a weak negative correlation between year of birth and anxiety/depression at follow-up, and a very weak positive correlation between birth year and gender, there were no significant correlations between birth year and other variables. Medication was associated with both anxiety/depression symptoms and attention problems at T1 and T2, but more strongly at T2. On the other hand, therapy was more strongly correlated with therapy at T1 compared to T2, and medication and therapy was positively correlated. Self-esteem was non-significantly associated with medication and therapy.

### Structural equation modeling

Structural equation modeling was used to assess the effect of self-esteem on the stability of emotion problems and attention problems in the sample. In structural equation modeling, it is possible to combine latent factor analysis with standard regression analyses using sum scores, as well as many other modeling features [35]. In the present study, a semi cross-lagged model was defined, where each type of symptoms at follow-up were regressed on the other type of symptoms, as well as on their same type of symptoms at baseline. Also, to assess the effect of self-esteem on changes in symptoms from baseline to follow-up, a latent construct of the four self-esteem items at baseline was included as a predictor of symptoms at follow-up, and covariates were freed between self-esteem and the two symptoms-measures. A covariate was also freed between the two types of symptoms at baseline and the residuals at follow-up.

**Fig. 2:**
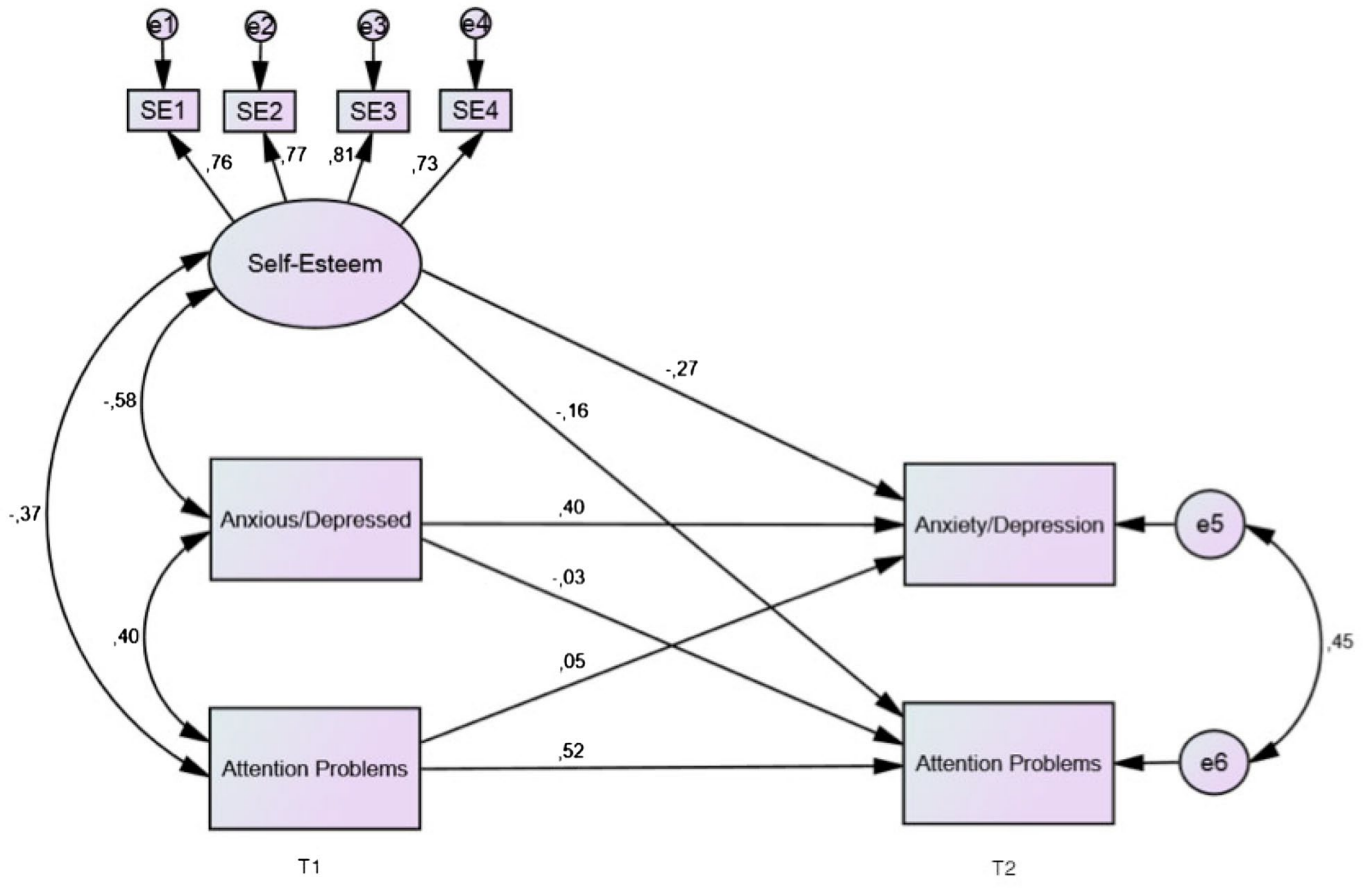
Cross-lagged panel model of self-esteem, attention problems, and symptoms of anxiety/depression at baseline (T1) and follow-up (T2). One-headed arrows illustrate regression effects; two-headed arrows illustrate correlations. The cross-lagged paths between Anxious/Depressed and Attention Problems were nonsignificant; all other effects and correlations were significant (p < .05)

The path model was tested in AMOS Version 22 for potential correlations and cross-lagged paths (see Fig. 2), using maximum likelihood estimation Missing data was not imputed or estimated, only subjects with responses at baseline and follow up were included in the longitudinal analyses. The model had good fit with the data: *χ* ^2^ (16, N = 717) = 77.07, *p* < .001, CFI = .965, TLI = .920, RMSEA = .073. In the model, there was a high negative correlation between self-esteem and anxiety/depression at baseline (*β* = − .58, *p* < .01), as well as between self-esteem and symptoms of attention problems (*β* = − .37, *p* < .01). However, the correlation was stronger between self-esteem and symptoms of anxiety/depression. Furthermore, the stability over time of symptoms of both anxiety/depression (*β* = .40, *p* < .01) and attention problems (*β* = .52, *p* < .01) was relatively high, controlled for each other at identical measure points.

Our main hypothesis was related to the influence of self-esteem on change in levels of symptoms over time. Results showed that high self-esteem at baseline predicted a reduction in symptoms of both anxiety/depression (*β* = − .27, *p* < .01) and attention problems (*β* = − .16, *p* < .01) at follow-up. These results support the assumption self-esteem is protective towards the development of both emotional problems and attention problems. Moreover, the difference between these paths was not significant (*z* = 1.72, *p* = .08, two-tailed test).

Finally, when we controlled for gender, medication and therapy at T1 in the model, it did not affect the longitudinal findings in any substantial manner. The self-esteem → anxiety/depression path weakened from *β* = − .27 to *β* = − .23, whereas the self-esteem → attention problems path remained unchanged at *β* = − .16. Model fits were good: *χ* ^2^ (27, N = 717) = 85.26, *p* < .001, CFI = .972, TLI = .931, RMSEA = .055.

## Discussion

In the present study, we assessed the longitudinal relationship of self-esteem and symptoms of anxiety/depression and attention problems among adolescents. In contrast to previous studies, we examined the role of self-esteem in the development of anxiety/depression symptoms and attention problems in a clinical psychiatric sample of adolescents, with particular focus on the relationship between self-esteem and attention problems.

First, and cross-sectionally, results showed that self-esteem was negatively related to symptoms of depression/anxiety and attention problems in our clinical sample of adolescents. These findings are consistent with previous studies on depression/anxiety, attention problems, and self-esteem conducted in both clinical samples and community samples [17–20, 24, 36, 37]. Also, and as expected, symptoms of depression/anxiety were positively related to attention problems at both baseline and follow-up. Symptoms of anxiety/depression and attention problems were moderately stable over time, more so for attention problems than for anxiety/depression. Second, and in accordance with our main hypotheses, the path model showed that high self-esteem at baseline predicted a dampening in symptoms of *both* anxiety/depression and attention problems at follow-up. Notably, these effects remained highly significant after we controlled for gender, medication, and therapy.

The present results indicate that self-esteem protects against the development of attention problems and anxiety/depression among adolescents under treatment for mental health problems. Thus, self-esteem may be of clinical relevance, despite not being regarded as a clinical term. Self-esteem may tap into positive aspects of one’s self, and as such constitute a source for resilience. When adolescents are under treatment, it may be fruitful for the clinician to focus on the strengths and qualities of the patient, in order to build a solid foundation for further treatment. A positive evaluation of the self may counteract symptoms of mental health problems in adolescence, although the actual mechanism for this is unclear. However, the protective effect of self-esteem found in the present study may in part be explained by how self-esteem affects stress coping, which is partly related to self-efficacy [21]. Studies have shown that high self-esteem acts as a buffer under stress, hence reducing harmful effects of stress on mental health [38]. When an individual is exposed to stress, he/she will utilize different strategies, or coping mechanisms. Lazarus and Folkman [39] described coping mechanisms as cognitive and behavioral efforts that individuals apply in order to tolerate, escape or minimize the effects of stress [40]. They described two main strategies: (a) the active problem-solving strategy, and (b) the avoidant strategy. Problem-solving strategies are considered functional because they allow confrontation of the problem, processing of the stress, and thus functional adaption. Avoidant strategies, on the other hand, are considered dysfunctional [41]. A possible explanation for this is that avoidant strategies disable processing of, and adaption to, the problem. It has been shown that individuals with low self-esteem often adopt passive-avoidant coping styles focused on emotions, whereas individuals with high self-esteem will adopt active problem focused coping strategies [38, 41]. Also, some studies have shown that high self-esteem is associated with persistence when facing adversities [1]. These are possible mechanisms/explanations for how high self-esteem can act as a resilience factor against long-term internalizing problems, such as depression and anxiety.

Furthermore, self-esteem was also negatively associated with attention problems. A study on adults with ADHD found that these subjects favored the use of maladaptive coping strategies [42]. Furthermore, attention problems were negatively associated with seeking advice and support from others. It is likely that maladaptive coping strategies and lack of social support in problem solving may lead to reduced self-esteem. Some researchers have suggested that children with attention problems may struggle to attend to social cues that allow them to engage in successful social interactions [43]. Tseng and Kawabata [44] suggested that problem with behaviors such as sharing and listening, could by others be perceived as inattentive or unsupportive behavior, which in turn may lead to poor peer liking. Negative peer feedback and rejection is likely to cause a negative sense of self, which in turn may lead to an increase in maladaptive behavior. Adolescents rejected by peers might also miss out on practicing reciprocal social interactions. Stenseng, Belsky, Skalicka, and Wichstrøm [45] found that lack of social belonging led to increased symptoms of hyperactivity-impulsivity and inattentiveness. It is possible that this manifests as a vicious cycle, where attention problems lead to peer rejection and low self-esteem, which in turn increases symptoms. If self-esteem is a protective factor against symptoms, appraising self-esteem may affect long-term outcome of ADHD in adolescents.

Symptoms of both anxiety/depression and attention problems were moderately stable over time. Stability of attention problems was higher than for anxiety/depression, as expected. This may be due to the neurobiological nature of attention problems [46]. Furthermore, whereas ADHD is mostly attributed to genetic makeup [47], symptoms of anxiety and depression are considered to be more dependent upon contextual factors and life circumstances. This may partly explain why attention problems were more stable over time than emotional problems. Although the stability of attention problems was relatively high, even greater stability of symptoms may have been expected within a clinical population. The decrease in symptoms shows that—despite a strong genetic disposition in the development of ADHD—self-esteem may act as a resilience factor against future symptoms of both attention problems and anxiety/depression. This emphasizes the gravity of self-esteem and indicates that self-esteem is of importance, also in a clinical setting. When self-esteem also inflicts on the development of attention problems, this indicates that clinicians should take a holistic view on their patients’ challenges, and be carful to tie diagnoses to their patients’ problems at an early stage in the treatment process. Symptoms are highly overlapping, so treatment of one category of mental health problems will often also reduce symptoms of another category of problems.

The present study has some limitations. First, analyses were based on symptoms of mental health problems, not diagnoses. Hence, findings cannot be directly transferred to adolescents with anxiety disorders, depressive disorders, or ADHD. However, within a clinical population, it is likely that a considerable part of the subjects with symptoms of attention problems will have a diagnosis of ADHD. Similarly, subjects with symptoms of anxiety and depression in a clinical sample are likely to be diagnosed with anxiety and/or depression. It is also possible that the prospective effects on the anxiety/depression scale found in the present study, may have turned out differently if we had measured anxiety and depression symptoms separately. Second, subjects diagnosed with ADHD, anxiety or depression may have received medical treatment during the study period, which may have reduced or altered symptoms. Third, a short version of the RSES was used in the present study. Although this may have affected self-esteem scores, the four-item version correlates highly with the original scale, and has demonstrated validity as a measure of self-esteem [48, 49]. Finally, as this study was performed in a clinical population, results are not representative for the general population.

## Conclusions

The present study demonstrates that clinically assessed adolescents with high self-esteem suffer fewer symptoms of anxiety/depression and attention problems over time, indicating that self-esteem acts as a resilience factor against such symptoms. Hence, the present study highlights the importance of self-esteem in a clinical setting, and that addressing self-esteem in clinical practice may affect the long-term outcome of both anxiety/depression symptoms and attention problems among adolescents.

## Abbreviations

ADHD: attention deficit/hyperactivity disorder
CAP: The Health Survey in the Department of Child and Adolescent Psychiatry
YSR: Youth Self-Report
RSES: Rosenberg Self-Esteem Scale
SPSS: Statistical Package for the Social Sciences
AMOS: analysis of moment structures
